# Bridging the Age Gap: Failure-to-Rescue rates in patients undergoing Radical Cystectomy

**DOI:** 10.21203/rs.3.rs-11347234/v1

**Published:** 2026-10-30

**Authors:** Alaa Balaghi, Nadim Alame, Mohammad Ali Mazloum, Dany Eid, Sofia Abdul Khalek, Albert El Hajj

**Affiliations:** American University of Beirut Medical Center; American University of Beirut Medical Center; American University of Beirut Medical Center; American University of Beirut; American University of Beirut; American University of Beirut Medical Center

**Keywords:** Failure to rescue, radical cystectomy, bladder cancer, postoperative complications, 30 day mortality, frailty, risk stratification, surgical outcomes, patient safety, quality metrics

## Abstract

**Purpose:**

Radical cystectomy (RC) for bladder cancer carries substantial morbidity, with 30-day mortality rates often reported at 2–3%. “Failure-to-rescue” (FTR) – defined as death following one or more major postoperative complications – has emerged as a key quality metric in surgery. We used the ACS-NSQIP dataset to quantify 30-day FTR after RC and identify its predictors.

**Methods:**

We retrospectively analyzed ACS-NSQIP (2019–2022) to identify 11,062 adults undergoing elective RC for malignancy. Patients were separated into age groups by percentiles, < 62 (25th percentile), 62–76 (25th – 75th percentile), and > 76 (75th percentile). We defined major complications as Clavien Dindo grade ≥ III. FTR was defined as 30-day mortality among patients with ≥ 1 major complication. We performed multivariable logistic regression to identify independent predictors of FTR while adjusting for patient demographics and comorbidities.

**Results:**

The 30-day mortality was 1.4%, with 10.5% sustaining ≥ 1 major complication. FTR occurred at a rate of 9.51% overall, with older age groups (> 76) showing higher FTR rates (16.06%) while lower age groups were much lower, age < 62 (5.65%) and age 62–76 (7.09%). Multivariable analysis identified older age (> 76 years) associated with significantly higher FTR (OR 2.884, p-value = < 0.001) while middle age groups did not see a significant increase of FTR after developing major complications (OR 1.234, p-value = 0.504).

**Conclusions:**

Older patients undergoing cystectomy were associated with greater failure-to-rescue rates. These findings complement prior studies showing that hospital and patient factors influence FTR. Interventions to improve early complication recognition and rescue may reduce FTR and improve survival after RC.

## Introduction

Bladder cancer is among the most common cancers globally and ranked as sixth in incidence in the United States. In a recent study done by Teoh et al assessing the global ten-year trends in bladder cancer, bladder incidence has been shown to be decreasing across many regions while showing discordance in mortality rate trends(1). Bladder cancer varies between muscle invasive(MIBC), which constitutes around 25% of the cases, or non-muscle invasive(NMIBC), which are about 75% of the cases(2). In the MIBC subtype, the gold standard treatment is neo-adjuvant systemic treatment followed by radical cystectomy(RC) when it is confined to the bladder. Surgical complications are common in this highly morbid procedure and include gastrointestinal complications occurring in about 20% of the RC cases, followed by infectious complications(17%)(3). For this reason, patient selection and early detection and management of complications is paramount.

Failure to rescue(FTR), first coined by Silber et al. in 1992, is the mortality rate among patients who develop complications(4). A low FTR signals effectiveness at detecting and managing complications. Silber’s group and others showed that differences in postoperative mortality across hospitals were explained more by variation in FTR than by variation in complication rates(5). When comparing high mortality hospitals to low mortality ones, the number of complications did not differ but the FTR rate was nearly double(21% vs 12%)(5). In addition, elderly patients present with more co-morbidities and are more prone to complications with higher risk of mortality than their younger counterparts(6). To date, no national-level study has specifically examined age-related differences in failure-to-rescue after RC.

Our study aims to assess the 30-day failure to rescue rates in patients undergoing radical cystectomy for muscle-invasive bladder cancer, stratified by age groups, using the American College of Surgeons National Surgical Quality Improvement Program(ACS-NSQIP) database.

## Methods

### Data Source and Patient Selection

This study is a retrospective cohort study using the ACS-NSQIP Participant Use Files(2019–2022). Patients are followed for 30 days after surgery, however complications or deaths beyond 30 days are not captured in NSQIP(7). Using Current Procedural Terminology(CPT) codes 51570, 51575, 51580, 51585, 51590, 51595, 51596, 51597, 51999, we identified all patients who underwent radical cystectomy, limited to years 2019–2022 with a total of 11,062 RC cases meeting inclusion criteria. Patients less than 18 years old and cases with missing key data were excluded. Each patient’s age at surgery was recorded, and patients were stratified into three groups based on 25th and 75th percentiles: less than 62, 62 to 76 years, and greater than 76 years of age. This stratification was chosen to facilitate comparisons across a younger group, an intermediate reference group, and a clearly older group. This also mirrors thresholds used in prior RC outcome studies for “older” patients(8).

### Definitions and Outcome Measures

Our primary outcome was failure-to-rescue(FTR) within 30 days. We defined FTR as 30-day postoperative mortality in patients who experienced at least one major complication(9). We considered major complications as Clavien–Dindo grade III or IV, which are complications that require surgical or procedural intervention(grade III) or life-threatening complications requiring ICU care(grade IV)(10). Patients who died without a major precedent event, were not considered FTR and were analyzed separately. Secondary outcomes included overall 30-day mortality, complication rates and severity distribution, and FTR rates within each age group. To determine complication severity, we determined each patient’s highest Clavien–Dindo grade complication within 30 days. Based on the NSQIP definitions and requirements for intervention we mapped all complications recorded in NSQIP to a Clavien grade as reported in Supplementary Table 1(11).

Baseline characteristics such as demographics, body mass index(BMI), smoking status, comorbidities, American Society of Anesthesiologists(ASA) physical status, and receipt of neoadjuvant chemotherapy were all extracted and analyzed.

### Statistical Analysis

Patient characteristics and outcomes across the three age categories were compared through univariate analyses, we also used Chi-square tests for categorical variables, and one-way ANOVA and Kruskal–Wallis tests for continuous variables. A p-value < 0.05 was considered statistically significant.

The FTR rate for each age group was calculated by: number of FTR events in that age group divided by number of patients with one or more Clavien III–IV complication in that age group × 100%. We constructed a bar chart(Supplementary Fig. 1) to visualize differences in FTR percentage by age category.

For the primary analysis, a multivariable logistic regression model was used to assess FTR rates across age groups, adjusting for covariates that can potentially influence rescue, such as sex, race, BMI, ASA class, smoking status, and comorbidities such as hypertension, diabetes, COPD, congestive heart failure, need for dialysis, and recent chemotherapy. An enter method combined with backward stepwise elimination, where variables with p < 0.10 were retained in the final model. Results are presented as odds ratios(OR) with 95% confidence intervals. P-value < 0.05 was considered statistically significant. All analyses were conducted using SPSS version 31.0.

Because this study used de-identified, publicly available NSQIP data, it was deemed exempt from institutional IRB review.

## Results

### Patient Characteristics

11,062 radical cystectomy patients were included, with a median age of 70 years and age range 18–89. 25% of patients were younger than 62 years(n = 2,802), 50% were 62–76(n = 5,414), and 25% were older than 76(n = 2,846)(Table 1). Older patients(> 76) had a significantly smaller proportion of females (20.0% vs 28.9% in < 62, *p* < 0.001), more medical comorbidities(77.1% ASA class 3 and 9.8% ASA 4–5 vs 65.6% and 5.1%, *p* < 0.001), more hypertension(69.8% vs 39.3% in < 62, *p* < 0.001), and slightly lower BMI on average(27.4 vs 29.3, *p* < 0.001).

Only 37.4% of those younger than 62 and 37.2% of 62–76 patients received chemotherapy within 90 days pre-operatively, versus 24.6% of > 76 patients(*p* < 0.001), which likely reflects more frequent cisplatin ineligibility or caution in the elderly. Other variables(race distribution, smoking rates, etc.) also varied with age(all *p* < 0.001)(Table 1).

### Complications and Mortality

52.9% of patients experienced at least one postoperative complication of any Clavien–Dindo grade within 30 days, with the majority being minor or moderate. Grade II was the most common severity with 36.4% of all patients having a Clavien grade II complication as their worst event. Whereas Grade I was the highest complication in 5.6%, grade III in 5.3%, and grade IV in 4.2% of patients and the remaining 1.4% being grade V which is death. Older patients also had slightly higher rates of severe complications, for instance 4.7% of > 76 patients had a grade IV complication versus 3.5% of patients < 62(*p* < 0.001). In addition, 42.3% of > 76 patients had no complications, compared to 49% in younger groups. The 30-day mortality for the entire cohort was 1.4%, with the majority, 94.7%, occurring in patients who had at least one postoperative complication.

Supplementary Table 3 showcases the maximum Clavien grade by survival status at 30 days. The patients who survived were distributed as 10.9% grade I, 70.6% grade II, 10.4% grade III, and 8.1% grade IV. On the other hand, among the patients who died (FTR cases), 73.6% experienced a grade IV complication,21.5% had grade II, 3.5% had grade III, and 1.4% had a grade I event. These differences were significant with *p* < 0.001.

### Failure-to-Rescue

Overall failure-to-rescue (FTR) for the cohort was 9.51%, meaning that if a patient experienced a major complication after RC, their chance of surviving it was about 90%. Supplementary Fig. 1 showcases FTR when stratified according to age, with patients > 76 years having an FTR rate of 16.06%, significantly(*p* < 0.001) higher than middle(7.09%) and younger(5.65%) age groups. When looking at FTR by complication(Supplementary Table 2), cardiovascular incidents were the most morbid.

### Multivariable Analysis of FTR Predictors

Table 2 presents the univariate and multivariate logistic regression results for failure-to-rescue. In the univariate model, using age < 62 as the reference, the 62–76 age group had an OR of 1.273 which was not significant(*p* = 0.434). In contrast, the > 76 group had an unadjusted OR of 3.192 which was significant(*p* < 0.001).

After adjustment for differences in baseline characteristics and comorbidities, OR for age > 76 was 2.884(*p* < 0.001). Ages 62–76 again showed no significant association (OR 1.234, *p* = 0.504). Using ASA 4–5 (highest risk category) as the reference, patients with ASA 2 had an adjusted OR of 0.131(*p* < 0.001) and ASA 3 had OR 0.428(*p* = 0.001). This signifies that as ASA class increases FTR increases, with ASA class 4 being 7.6 times more likely to suffer FTR than ASA class 2. Other patient factors were not statistically significant independent predictors in the final model. The model’s AUC was 0.690, indicating moderate discrimination for predicting FTR, which reflects the challenge of predicting rare events like death after complications.

## Discussion

In this large, contemporary analysis of radical cystectomy outcomes according to age, we found that advanced patient age is strongly associated with failure-to-rescue from postoperative complications. We found that RC patients older than 76 years had a FTR rate of nearly three times compared to patients under 62, even after adjusting for differences in comorbidity. However, patients in their 60s and early 70s had FTR outcomes comparable to much younger patients. This suggests that beyond a certain age threshold, the ability to survive major surgical complications drops significantly.

The literature aligns with our findings that elderly patients are at elevated risk of FTR. Wells et al conducted a retrospective national cohort study to assess the effect of age, frailty, and comorbidities on failure to rescue rates of patients undergoing surgery for gastrointestinal cancer. Their cohort included 21,729 patients and similarly found that with every increase of 5 years of age, the odds ratio of 90-day morbidity was 1.40 (95% CI 1.34–1.46) on adjusted analysis (12).

Our overall 30-day mortality after RC was 1.4%, which is considered on the low end of previously reported ranges, with most large series reporting 2–3% 30-day mortality(8). This is likely due to improvements in perioperative care and the influence of case selection, selecting the fittest elderly patients.

Most deaths in our cohort were associated with severe (Clavien IV) complications rather than technical complications that may require re-operation (Clavien III). This suggests that what often leads to mortality after RC is the physiological aftermath, such as infections or organ dysfunction, rather than surgical complications. Henneman *et al.* in a study of colorectal surgery found that high-mortality hospitals had a threefold higher FTR rate compared to low-mortality hospitals, even though complication rates differed little(13).

Our results showcase that patient factors influenced FTR in this cohort rather than intraoperative and hospital factors. All the modifiable variables we examined were not significantly associated with FTR after adjustment. Instead, the only predictors of FTR were age and ASA. This shows that certain patients, such as high frailty or ASA4 patients, are inherently at higher risk regardless of how well equipped a hospital is. Zakaria et al reported that high-volume centers had significantly lower 30-, 60-, 90-day mortality after RC than low-volume centers (8). This might be due to better protocols for early recognition of complications, availability of expert multidisciplinary teams, and ICU resources, all of which improve rescue success. Another study by Ghaferi et al. found that variability in mortality was due to FTR differences attributable to hospital factors, not complication incidence(14). We could not directly assess hospital-level effects in our analysis due to NSQIP’s lack of hospital identifiers. However, all patients were treated at institutions that participate in NSQIP, which have somewhat higher quality standards.

As for our clinical implications, first we highlight the need for vigilant postoperative care and rapid intervention in elderly RC patients. Enhanced Recovery After Surgery (ERAS) protocols, though primarily aimed at reducing complications, may also indirectly improve rescue by maintaining better baseline function so the patient is in a stronger position to withstand complications. We suggest intensification of surveillance in the first 30 days for older patients, including more frequent follow-up calls or clinic visits in the early discharge period so that fast evolving complications are caught early. Second, these results should not be misinterpreted as advocating against surgery in older patients. On the contrary, given that many older patients in our cohort did survive, RC can be curative and beneficial even at advanced ages with proper selection. Thus, our analysis suggests that age by itself should not be an exclusion criterion, rather a risk stratifier that should trigger additional resources. Third, failure-to-rescue as a quality metric in urology could help identify gaps in care. Some studies have noted that focusing only on complication rates could be misleading, as two hospitals can have equal complication rates, but one saves patients with complications at a higher rate, and thus provides a higher quality of care(4). Urologic surgery departments could learn from experiences in general surgery, where raising the alarm early for a deteriorating patient is encouraged(15). This ensures rapid availability of surgeons and intensivists when complications occur, and encourages conducting mortality reviews specifically asking, “Was this death preventable with better rescue?”.

### Limitations

This study however is not without limitations. First, the analysis is retrospective and observational, which limits causal inferences. The use of NSQIP means we have limited confounders, such as lack of clinical stage, and only capture outcomes up to 30 days, with many complications occurring up to 90 days postop (16). Studies have cited 90 day mortality as high as 7.5% (8), thus our FTR measure likely underestimates absolute failure-to-rescue rates. Third, the lack of direct cause of death in NSQIP data limits our ability to introspect into which types of complications are less rescuable in the elderly. Prior studies suggest infectious and cardiopulmonary complications are the leading causes of death after RC(8), aligning with our finding of prevalent Clavien IV events. The cohort from NSQIP centers may not be fully representative of all hospitals, since they might be larger or more academic on average. Finally, our study primarily focused on the patient age factors, with interventions not being evaluated. Thus, even though associations can be made, we cannot directly prove what interventions would reduce FTR in the elderly, which would require targeted studies or quality improvement trials.

Despite its limitations this study has several strengths. We used a large, recent, multi-center dataset with standardized outcomes, which enhances the reliability of FTR measurement. Age stratification provides novel insight into how patient-specific factors affect perioperative quality of care. Our findings were also corroborated with established literature on FTR in other contexts, which gives face validity to our conclusions.

## Conclusion

To conclude, this study showed that patient age is an important determinant of FTR after RC, with patients over 76 years old significantly less likely to survive a major postoperative complication than younger patients. In terms of clinical practice and healthcare quality improvement, the need for intensified postoperative surveillance and aggressive management of complications in elderly patients undergoing RC appears to be paramount. Advanced age should trigger heightened readiness from healthcare teams to intervene. It should not serve as a barrier to performing RC, but rather a warning to optimize our care, thus bridging the age gap in bladder cancer surgery outcomes.

## Supplementary Material

This is a list of supplementary files associated with this preprint. Click to download.

• SupplementaryTablesWJU.docx

## Figures and Tables

**Table 1.** Patient demographics divided by age cohort

		Age Group			
		< 62 (2802)	62–76 (5414)	> 76 (2846)	p-value
**Sex**	**Female**	811 (28.9%)	1051 (19.4%)	570 (20.0%)	< 0.001
	**Male**	1991 (71.1%)	4362 (80.6%)	2276 (80.0%)	
**Race**	**White**	2009 (71.7%)	4099 (75.7%)	2138 (75.1%)	< 0.001
	**Non-White**	261 (9.3%)	343 (6.3%)	142 (5.0%)	
	**Unknown**	532 (19.0%)	972 (18.0%)	566 (19.9%)	
**ASA Class**	**2**	788 (28.2%)	1042 (19.3%)	373 (13.1%)	< 0.001
	**3**	1835 (65.6%)	4002 (74.0%)	2191 (77.1%)	
	**4 + 5**	144 (5.1%)	358 (6.7%)	278 (9.8%)	
**BMI**		29.3 (± 6.7)	28.7 (± 5.6)	27.4 (± 4.7)	< 0.001
**Hypertension**		1101 (39.3%)	3288 (60.7%)	1987 (69.8%)	< 0.001
**CHF**		29 (1.0%)	101 (1.9%)	102 (3.6%)	< 0.001
**Diabetes**		422 (15.1%)	1194 (22.1%)	561 (19.7%)	< 0.001
**COPD**		101 (3.6%)	391 (7.2%)	213 (7.5%)	< 0.001
**Preop Dialysis**		34 (1.2%)	43 (0.8%)	25 (0.9%)	0.163
**Smoker within year**		884 (31.5%)	1098 (20.3%)	218 (7.7%)	< 0.001
**Chemotherapy within 90 Days of OR**		1049 (37.4%)	2013 (37.2%)	699 (24.6%)	< 0.001

**Table 2:** Unadjusted and adjusted odds ratios for failure-to-rescue by age group. Age > 76 was the only age category associated with significantly increased FTR odds. (Multivariate model adjusted for sex, BMI, race, ASA class, hypertension, CHF, diabetes, COPD, smoking, preop dialysis, and neoadjuvant chemotherapy; only age and ASA were significant.)

Age Group	Unadjusted OR (95% CI)	p-value	Adjusted OR (95% CI)	p-value
< 62 years	Reference	–	Reference	–
62–76 years	1.273 (0.695–2.332)	0.434	1.234 (0.666–2.289)	0.504
> 76 years	3.192 (1.790–5.693)	< 0.001	2.884 (1.558–5.338)	< 0.001

## Data Availability

Data sharing is not applicable to this article as no new data were created or analyzed in this study.
